# Personalizing the treatment of head and neck cancer in vitro: The 3D-OTC model

**DOI:** 10.1186/s12916-026-04883-z

**Published:** 2026-04-25

**Authors:** Fabian Stögbauer, Tobias Weiser, Ali Bashiri Dezfouli, Johannes Wirth, Luca Engelmann, Jan Budczies, Iordanis Ourailidis, Melanie Boxberg, Katharina Pigorsch, Benedikt Schmidl, Nicole Strittmatter, Katja Steiger, Carolin Mogler, Barbara Wollenberg

**Affiliations:** 1 Institute of Pathology, School of Medicine and Health, Technical University of Munich, Munich, Germany; 2 Department of Otolaryngology, Head and Neck Surgery, TUM Klinikum, Technical University of Munich, Munich, Germany; 3 Institute of Pathology, University Hospital Heidelberg, Heidelberg, Germany; 4 Faculty of Biosciences, University of Heidelberg, Heidelberg, Germany; 5 Department of Biosciences, TUM School of Natural Sciences, Technical University of Munich, Munich, Germany

**Keywords:** Head and neck cancer, in vitro model, morphology, tumor budding, microenvironment, longitudinal analysis, spatial transcriptomics, drug resistance testing

## Abstract

**Background:**

Treatment of head and neck squamous cell carcinoma (HNSCC) remains challenging and the survival rates of affected patients remain poor. A three-dimensional organotypic co-culture (3D-OTC) model where patient derived tumor tissue is cultured on human-derived fibroblasts (dermal equivalent, DE) was evaluated regarding its comparability to primary tumor tissue and its applicability in drug resistance testing.

**Methods:**

3D-OTC models were cultured from *n* = 10 HNSCC patients for up to 21 days. The growth pattern at the DE was compared to tumor budding of corresponding resection specimens. Furthermore, we immunohistochemically determined the immune cell infiltrate of primary tumor tissue and corresponding 3D-OTC models. Spatially resolved gene expression analysis (“Xenium in situ”) was performed for separate regions of interest within the 3D-OTC specimens and within primary tumor tissue. Up-regulated and down-regulated genes of the 3D-OTC samples were included in gene set enrichment analysis and up-regulated genes between invasive (invading the DE) and non-invasive tumor cells within the 3D-OTC samples were included in drug resistance testing using publicly available databases.

**Results:**

The growth pattern observed at the DE was associated with tumor budding in primary tumor tissue. The density of CD3-/CD20-/CD56-positive cells was lower in 3D-OTC samples compared to primary tumor tissue. No such changes were observed for CD68-positive cells and no significant changes in the density of the immune cell infiltrate were detected during the cultivation period. The centroids and dispersion of the gene expression of the 3D-OTC samples did not differ from the corresponding primary tumor tissue. The regions of interest within the 3D-OTC samples showed distinct functional states in gene set enrichment analysis. The comparison of genes up-regulated in invasive tumor parts of the 3D-OTC samples could explain resistance of tumor subclones to certain chemotherapeutics.

**Conclusions:**

The 3D-OTC model morphologically and transcriptomically resembles primary tumor tissue and its biology while preserving the tumor microenvironment. Furthermore, the 3D-OTC model allows the standardized evaluation of tumor tissue by the definition of transcriptomically separate regions of interest and thus, could help to evaluate the impact of personalized therapeutic interventions on the tumor and its microenvironment in vitro.

**Supplementary Information:**

The online version contains supplementary material available at 10.1186/s12916-026-04883-z.

## Background

Head and neck squamous cell carcinoma (HNSCC) is one of the most common cancers [1]. However, the treatment of HNSCC patients remains a challenge, especially in advanced tumor stages as responses to different treatment modalities are heterogeneous and difficult to predict due to a lack of reliable biomarkers [2, 3]. The assessment of the response to therapy could be facilitated by the establishment of preclinical models which would allow testing potential treatment options during the diagnostic workup [4]. These models should meet several requirements such as preserving the biological characteristics of the tumor as well as its microenvironment including immune cells [5]. Three-dimensional models appear to outperform two-dimensional models as they more closely mimic the exposure to oxygen, nutrients and drugs and maintain cell-cell interactions [6].

Recently, Engelmann et al. described a model called “3D organotypic tissue co-culture (3D-OTC)” which allows the ex vivo analysis of HNSCC samples and offers the advantages of a preserved morphology as well as tumor-immune-cell interactions and the possibility of therapeutic interventions [7]. The 3D-OTC model is derived from primary tumor tissue which is obtained during surgery and subsequently placed on a fibroblast layer for cultivation. Thus, this model might be able to simulate actual tumor stroma interactions [8]. The group demonstrated that the 3D-OTC model could be cultured for a time period of up to 21 days and could facilitate the cultivation of human papillomavirus-negative and -positive tumor samples [7]. The model was also shown to be suitable for the testing of therapeutic interventions like radiotherapy [7].

However, so far data are scarce describing the morphological concordance between the 3D-OTC model and primary tumor tissue, the exact cellular composition of the immune cell infiltrate especially over the time of cultivation and the applicability of modern “-omics” applications such as spatially resolved gene expression profiling.

Therefore, we aimed to further evaluate the 3D-OTC model regarding its morphological and transcriptomic comparability to primary tumor tissue. We also aimed to functionally characterize distinct regions of interest of the 3D-OTC model performing spatially resolved gene expression profiling. Differentially expressed genes should then be evaluated regarding their potential role in drug resistance.

Our study may help to establish the 3D-OTC model for deepening the understanding of molecular tumor progression and immunology as well as an in vitro platform for the standardized evaluation of personalized therapeutic options in HNSCC.

## Methods

### Patient cohort

Primary tumor tissue was obtained from *n* = 10 patients with squamous cell carcinomas of the head and neck after written informed consent was obtained. Patients underwent surgical resection at the Department of Otolaryngology, Head and Neck Surgery, TUM Klinikum, Technical University of Munich, Munich, Germany. Clinicopathological data of the cohort were extracted from patient records and pathology reports (Additional file 1: Table 1).

The patient cohort consisted exclusively of males with a median patient age of 62 years (interquartile range 14.5 years). Primary tumors were located in the larynx (*n* = 6, 60.0%), the oropharynx (*n* = 3, 30.0%) and the oral cavity (*n* = 1, 10.0%). According to the pathology reports among the oropharyngeal cancers *n* = 1 was immunohistochemically p16-positive and *n* = 2 were p16-negative. Regarding TNM staging *n* = 2 tumors (20.0%) were classified as pT1, *n* = 4 (40.0%) as pT2 and *n* = 4 (40.0%) as pT4, *n* = 3 tumors (30.0%) as pN0, *n* = 2 tumors (20.0%) as pN1, *n* = 1 tumor (10.0%) as pN2, *n* = 3 (30.0%) as pN3 and *n* = 1 (10.0%) as pNX. A total of *n* = 8 tumors (80.0%) were classified as moderately differentiated and *n* = 2 tumors (20.0%) as poorly differentiated. Most tumors were classified as conventionally keratinizing (*n* = 8, 80.0%) and *n* = 2 tumors (20.0%) as conventionally non-keratinizing.

Ethical approval was obtained from the Ethics Committee of the Technical University of Munich (15/21 S) and all analyses were conducted in accordance with the declaration of Helsinki.

### OTC generation

The culture of 3D-OTC samples was performed as previously described [7]. Briefly, dermal equivalents (DEs) consisting of human fibroblasts (NHDF (Normal Human Dermal Fibroblasts) cell line cells) were cultured on a viscose fiber fabric for up to 14 days (prior to surgery) in DE medium (DMEM low glucose (1 g/L, #21885108, Thermo Fisher, Waltham, MA, USA)/Ham’s F12 (#31765035, Thermo Fisher; DMEM/Ham’s F12 = 1:1), hydrocortisone (400 µg/L, #H0888, Sigma-Aldrich, St. Louis, MO, USA) and TGF-β (1 ng/mL) [7, 9]. Nonwoven viscose fabric (#Jettex 2005/45, 45 g/m² spunlace, 100% viscose; Orsa NW s.r.l., Gorla Minore, Italy) was used in this study. Circular specimens (11 mm diameter) were prepared using a puncher. The samples were subsequently sterilized by autoclaving prior to further experimentation. After surgical resection tumors were transported to the pathology laboratory and appropriate tumor tissue fragments with a diameter of around 0.3 cm were obtained by an expert pathologist (FS). The tumor tissues were then immediately placed on DEs and cultured in OTC medium, which was changed every third day (DMEM low glucose/Ham’s F12 (1:1)) supplemented with TGF-β (1 ng/mL), hydrocortisone (400 µg/L), aprotinin (6.5 µM, #A162.4, Carl Roth GmbH & Co. KG, Karlsruhe, Germany) and ascorbic acid (1 mM, #49752, Sigma-Aldrich, St. Louis, MO, USA). On days 7, 14, and 21 of cultivation, samples were harvested and immediately fixed in formalin or snap frozen for subsequent analysis.

### Histological analysis

Morphologic and immunohistochemical characteristics were analyzed on digitized hematoxylin and eosin (H&E) and immunohistochemically stained slides of the 3D-OTC specimens and the corresponding resection specimens [10]. For resection specimens the formalin-fixed paraffin-embedded (FFPE) blocks/slides with the highest amount of tumor tissue were selected for subsequent analysis. Digitalization was performed on an Aperio AT2 Digital Pathology scanner (Leica Biosystems, Nussloch, Germany). For all analyses areas of necrosis were excluded.

### Resection specimens

**Fig. 1:**
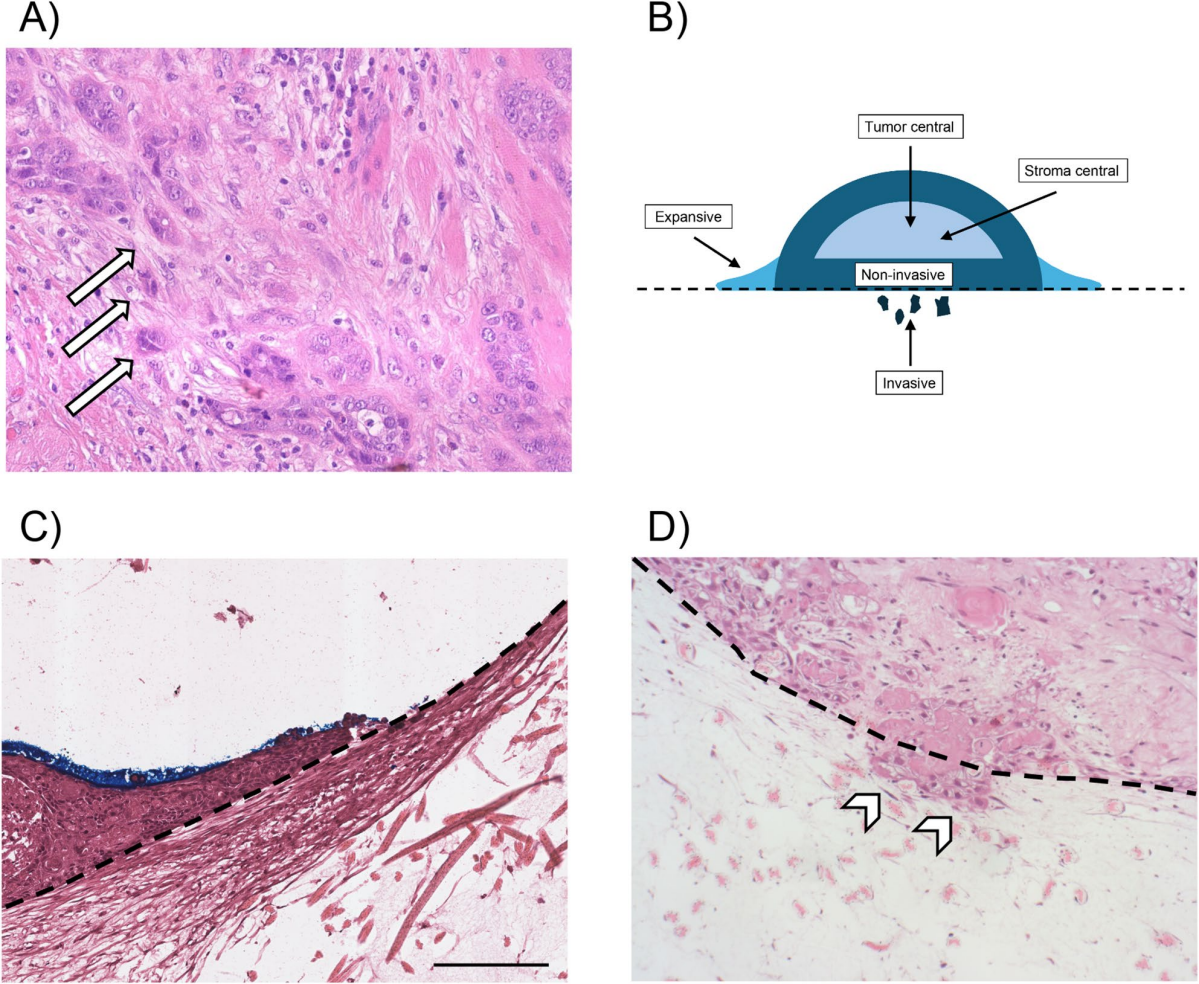
Illustration of the morphological analysis of resection specimens and 3D-OTC samples. Some exemplary tumor buds within the primary tumor are highlighted (arrows, **A**) (H&E, 200X). **A** Schematic illustrations of different regions of interest of the 3D-OTC samples. **B** Expansive growth-pattern at the DE. Tumor cells remain above the dashed line (H&E) (scalebar 200 μm). **C** Invasive growth pattern with infiltration of the DE (below the dashed line, arrowheads) (HE, 200X). **D** Illustrations (**C**) and (**D**) are derived from 3D-OTC numbers 6 (**C**) and 10 (**D**) cultured for 14 and 21 days, respectively

Tumor budding was evaluated in the resection specimens as previously described [11] (Fig. 1A). Briefly, tumor budding was defined as stroma-infiltrating single tumor cells or small tumor cell clusters consisting of up to four tumor cells dissociating from the main tumor mass. Tumor budding was evaluated in one high-power field (HPF; the one high-power field with the highest budding activity; one high-power field covered an area of 0.196 mm^2^). Absolute numbers for tumor budding were recorded.

### 3D-OTC model

FFPE blocks were cut into 2 μm thick sections, stained with H&E and analyzed under the microscope. Tumor budding was evaluated as described above. The immune cell infiltrate was determined as the proportion of the tumor area (without areas of necrosis) covered by immune cells.

According to Engelmann et al. the invasive front of the 3D-OTC model was evaluated for the growth pattern [7] (Fig. 1B-D). The “invasive” growth pattern was defined as tumor cells infiltrating the dermal equivalent. The “expansive” growth pattern was defined as tumor cells growing horizontally on the dermal equivalent without an infiltrative component. The “dormant” growth pattern was defined as vital tumors with no infiltrative component and no horizontal growth. All 3D-OTC specimens per case were evaluated and the presumed most aggressive growth pattern (invasive > expansive > dormant) was recorded for each case.

### Immunohistochemical analysis

For immunohistochemical analysis the slides were incubated with the primary antibodies (CD3, clone MRQ39, rabbit, Cell Marque, Rocklin, California, USA, 1:500, CD20, clone L26, mouse, Dako/Agilent, Santa Clara, California, USA, 1:2000, CD56, clone MRQ42, rabbit, Cell Marque, Rocklin, California, USA, 1:100, CD68, clone KP1, mouse, Dako/Agilent, Santa Clara, California, USA, 1:10000 and Ki67, clone ab16667, Abcam, Cambridge, UK, 1:50). Immunohistochemical staining for CD3 was performed on a Ventana BenchMark XT automated stainer (Ventana, Tucson, Arizona, USA) and for CD20, CD56, CD68 and Ki67 on a Bond RXm system (Leica, Wetzlar, Germany) (Additional file 1: Fig. S1, Additional file 1: Fig. S2A).

After digitization immunohistochemical slides were analyzed using QuPath (version 0.5.0) and applying the “positive cell detection” algorithm (Additional file 1: Table 2) [12]. On the slides of the resection specimens and the 3D-OTC models the outer contour of the tumor was annotated, immune cells within this border (intratumoral and stromal immune cells) were counted and positive immune cells per mm^2^ were recorded (Additional file 1: Fig. S3).

### Spatially resolved transcriptomic analysis

For the spatially resolved transcriptomic analysis *n* = 7 3D-OTC samples (fresh frozen tissue *n* = 3, FFPE *n* = 4) and *n* = 2 primary tumor tissue samples (FFPE) were available. The “Xenium in situ” platform (10x Genomics, Pleasanton, California, USA) was used as described in detail elsewhere [13]. Due to the targeted nature of the “Xenium in situ” platform a pre-designed multi-tissue and cancer panel consisting of *n *= 377 genes was applied (Additional file 1: Table 3) .

In brief, 5 μm thick sections were mounted on Xenium slides and dried for optimal adherence followed by deparaffinization, decrosslinking, probe hybridization, ligation and amplification steps. The preprocessed samples were then analyzed with the Xenium Analyzer (software version 1.5.1.2 and analysis version 1.5.0.3), stained with H&E according to the manufacturer’s protocol for post-Xenium stainings and digitized.

Subsequently, the H&E staining was removed and the slides were incubated with a pan-Cytokeratin monoclonal antibody (CKpan, clone MNF116, mouse, Invitrogen, Waltham, MA, USA, 1:200) on a Bond RXm system (Leica, Wetzlar, Germany) and also digitized.

For the evaluation of the results “Xenium explorer” (version 3.2.0) was used: H&E stained images, CKpan-images and DAPI-images were co-registered and annotated by an expert pathologist (FS) to obtain transcripts per area (Additional file 1: Fig. S4). The following regions of interest were annotated in 3D-OTC samples (Fig. 1B): non-invasive (defined as 1–2 layers of tumor cells at the base of the 3D-OTC model which did not extend beyond the borders of the original tumor tissue), invasive tumor cells (invading the DE), expansive tumor cells (defined as tumor cells growing above the DE from the hypothetical intersection of the convex tumor outline of the 3D-OTC model and the DE to the utmost lateral border), central tumor cells and central stroma cells (core of the 3D-OTC sample with a distance from the surface of the sample of at least around 100 μm) (Additional file 1: Fig. S4). In primary tumor tissue, all invasive tumor cell nests were analyzed and three regions of interest were defined: The outermost row of tumor cells in the invasive tumor cell nests, the remaining inner tumor cells in the invasive tumor cell nests and the peritumoral stroma (150 μm around the invasive tumor cell nests).

The transcriptomic readouts expressed as absolute counts could then be mapped to the annotated areas. Batch correction was conducted before downstream gene expression analysis [14]. Differentially expressed genes between all regions of interest were determined using DESeq2 normalizing for the area of gene detection and correcting the log fold changes [15, 16]. The genes were then included in gene set enrichment analysis using the MSigDB to functionally characterize the regions of interest [17–19].

### Evaluation of potential drug responses

To evaluate the therapy response of invasive tumor parts compared to non-invasive tumor parts we have conducted two web-based interrogations (the “HNCDrugResDb”, https://ciods.in/drug_resistant and “A database of genes related to platinum resistance”, https://ptrc-ddr.cptac-data-view.org/#) on March 20th 2026 [20, 21]. At the “HNCDrugResDb” we have conducted a “Drug Resistance Analysis” entering all up-regulated genes of invasive compared to non-invasive tumor areas, at “A database of genes related to platinum resistance” we have sequentially entered each up-regulated gene into the query field.

### Statistical analysis and software

R (version 4.4.1; R Core Team (2024). R: A Language and Environment for Statistical Computing. R Foundation for Statistical Computing, Vienna, Austria. https://www.R-project.org/) was used for statistical analysis. Spearman correlations were calculated to test for a correlation between the number of tumor buds in resection specimens and 3D-OTC samples and to calculate correlations between gene expression data and drug sensitivity and viability of cell lines. The Levene-Test was calculated to determine the equality of variances of the number of tumor buds and the immune cell infiltrate in the 3D-OTC samples. Fisher’s exact test was used for ordinally scaled data and the Mann-Whitney-U-Test for continuous data. A (global) chi-squared test was used to test for significance in the composition of the immune cell infiltrate. To determine the longitudinal differences of the specific immune cell composition we conducted pairwise chi-squared tests (Post-Hoc-tests). For dependent groups (primary tumor tissue and corresponding 3D-OTC samples) the Wilcoxon signed rank test was used. A permutational multivariate analysis of variance (PERMANOVA) was conducted to evaluate the similarity of the gene expression between the different regions of interest of the 3D-OTC samples. A paired PERMANOVA test was conducted for 3D-OTC samples and corresponding primary tumor tissue to evaluate the similarity of the gene expression of 3D-OTC samples and paired primary tumor tissue, respectively. Similarly, the dispersion of the gene expression of the regions of interest in the 3D-OTC samples and the primary tumor tissue was analyzed. In gene set enrichment analysis gene sets with an adjusted p-value < 0.25 were considered significant [17]. All tests were conducted two-tailed and *p* < 0.05 was considered as significant for all remaining analysis. The Benjamini-Hochberg procedure was used to correct for multiple testing.

The R packages used for statistical analysis and visualization are shown in Additional file 1: Table 4.

## Results

### Morphological analysis of 3D-OTC models

Table 1 provides an overview over the 3D-OTC samples evaluated. We successfully cultured a mean number of *n* = 9 (standard deviation 4) samples per case. An equal number of 3D-OTC models exhibited an expansive and an invasive growth pattern (50% each) whereas no dormant growth pattern was observed.

We tested whether the detection of an invasive growth pattern might be related to the number of 3D-OTC models cultured and thus, might be influenced by a sampling bias. However, we did not find an association between the growth pattern and the number of 3D-OTC samples cultured (*p* = 0.07). We observed significant differences in the variance of the number of tumor buds (variance: 2.93, 95% confidence interval 1.84–5.36, *p* = 0.026) but not for the immune cell infiltrate (variance: 6.24, 95% confidence interval 3.93–11.41, *p* = 0.129) of the cultured 3D-OTC samples.

**Table 1:** Clinicopathologic characteristics of the patients and 3D-OTC samples included in the study

3D-OTC	Age	Sex	Location	Timepoint of harvest	Growth pattern	Recurrence	Overall survival
1	37	male	larynx	d14	expansive	no	alive
2	70	male	oral cavity	d7, d14, d21	invasive	yes	alive
3	67	male	larynx	d14	expansive	no	alive
4	73	male	larynx	d7, d14, d21	expansive	no	alive
5	54	male	larynx	d7	expansive	no	dead
6	83	male	larynx	d7, d14, d21	invasive	no	alive
7	50	male	oropharynx (p16 negative)	d7, d14	invasive	no	dead
8	66	male	larynx	d7, d14, d21	invasive	no	alive
9	58	male	oropharynx (p16 positive)	d7, d21	expansive	no	alive
10	57	male	oropharynx (p16 negative)	d21	invasive	no	alive

IHC: Immunohistochemistry, d7/d14/d21: 3D-OTC sample cultivated for 7/14/21 days

### Correlation between tumor budding and the growth pattern

A median number of 1 tumor bud (interquartile range 0) was counted per case in the 3D-OTC samples and a median of 2 tumor buds (interquartile range 0) was counted in the resection specimens.

**Fig. 2:**
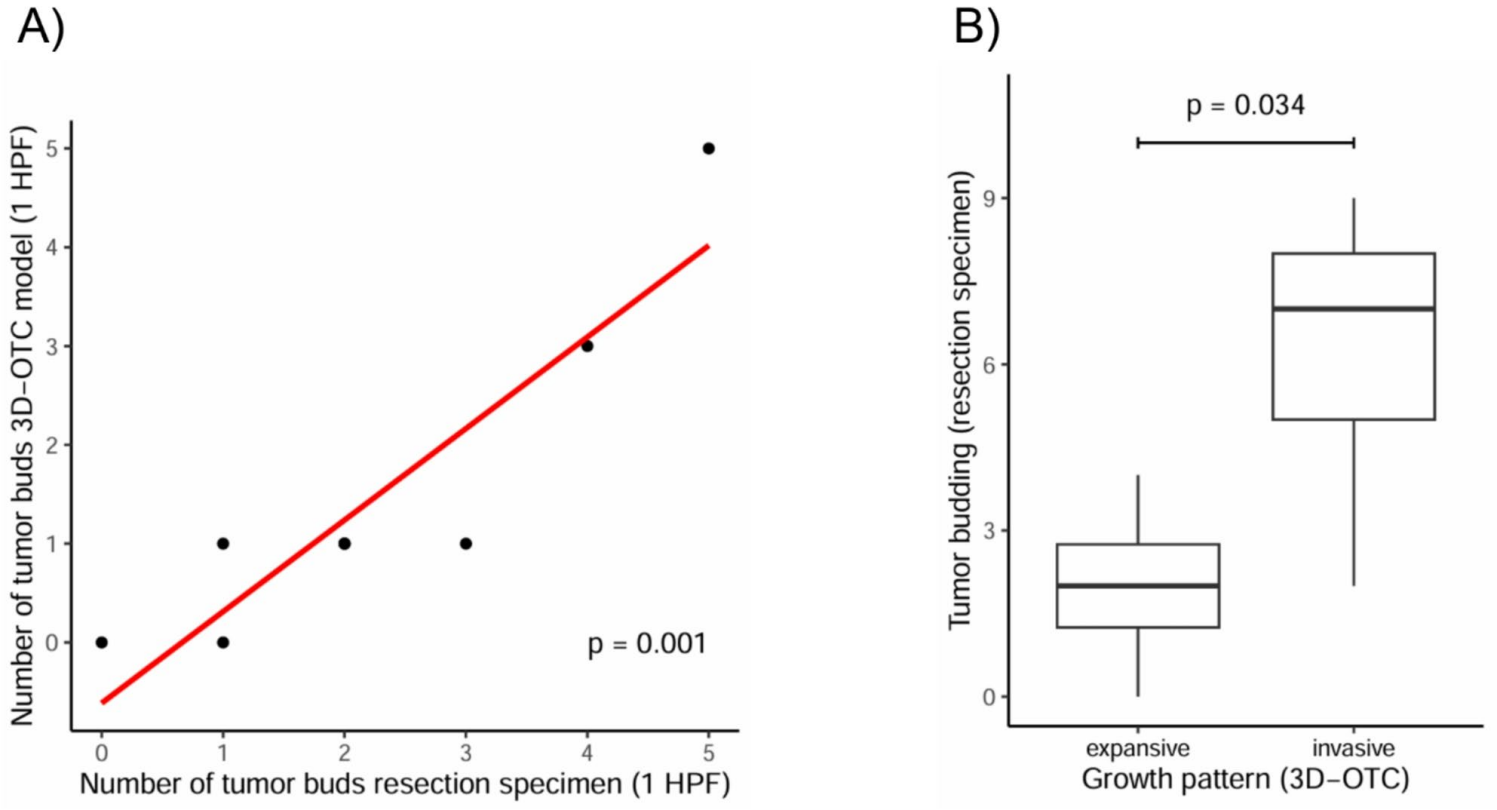
Comparison of tumor budding in resection specimens and 3D-OTC samples. Strong relationship between the number of tumor buds in resection specimens and 3D-OTC samples. **A** Association between the number of tumor buds and the growth pattern in 3D-OTC models. **B** Tumors with an invasive growth pattern in the 3D-OTC model had a higher number of tumor buds in the resection specimens. HPF: High-power field

We observed a strong relationship between the number of tumor buds in resection specimens and the 3D-OTC samples evaluating 1 HPF each (Spearman’s rho: 0.90, *p* = 0.001) (Fig. 2A).

We then tested for an association between the presence of tumor buds in resection specimens and the worst growth pattern observed in the 3D-OTC models. Tumors with an invasive growth pattern in the 3D-OTC model had a higher number of tumor buds in the resection specimens (median/interquartile range: invasive 7/3, expansive 2/1.5, *p* = 0.034, Fig. 2B).

When we correlated the expression of the proliferation marker Ki67 in the 3D-OTC samples and the growth pattern at the DE we could not detect significant differences (Additional file 1: Fig. S2B).

We could not observe a correlation between the growth patterns and lymphovascular invasion (*p* > 0.999) and perineural invasion (*p* = 0.546) in the resection specimens.

### Immune cell infiltrate at different time spans of cultivation

In *n* = 4 cases, 3D-OTC samples could be collected on days 7, 14 and 21 for longitudinal analysis of the immune cell infiltrate. In the remaining *n* = 6 cases longitudinal analysis could not be performed (not enough invasive tumor, necrosis, unsuccessful cultivation).

**Fig. 3:**
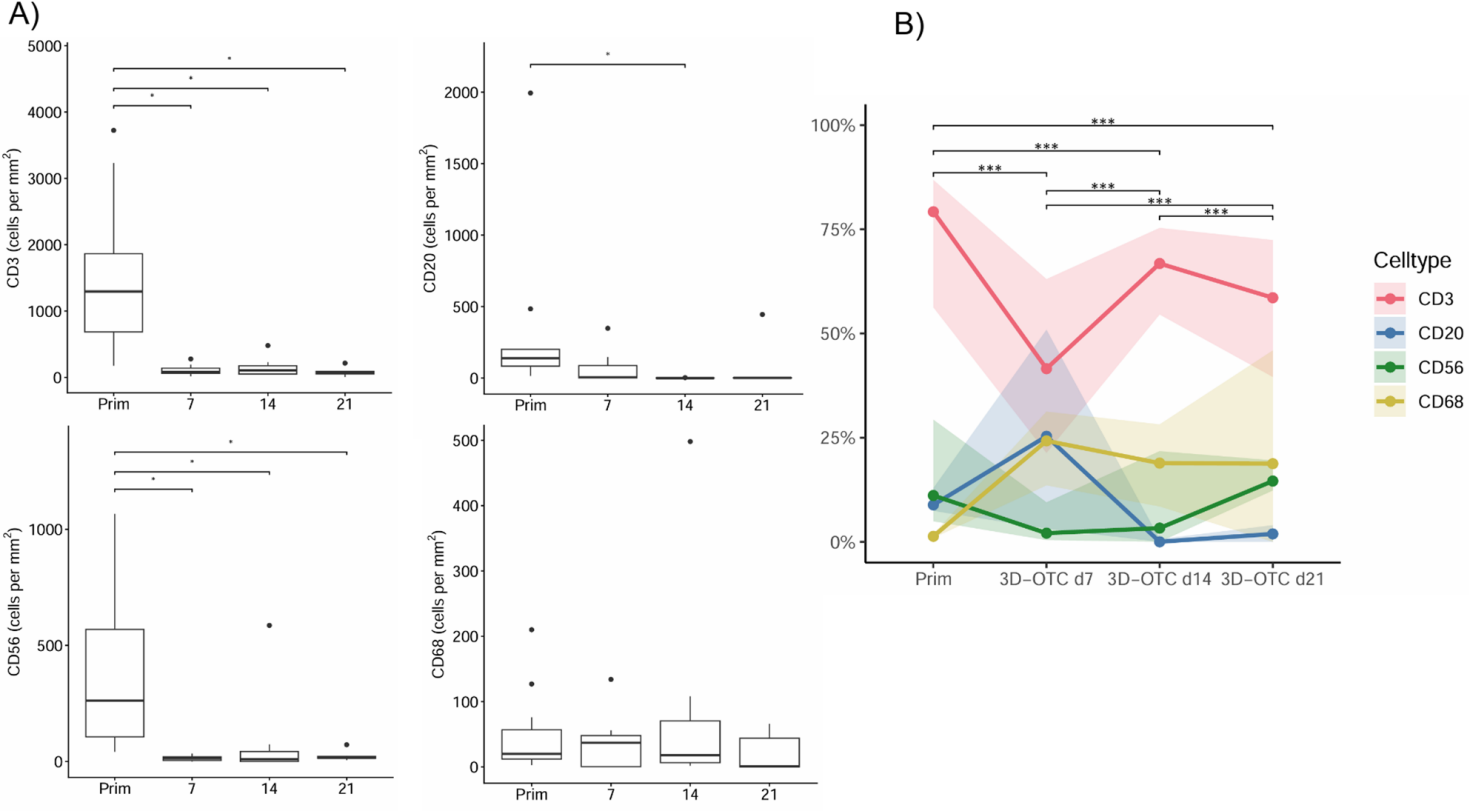
Results of the immunohistochemical analysis of the immune cell infiltrate. The density of CD3-/CD20-/CD56-/CD68-expressing cells is shown comparing primary tumor tissue with 3D-OTC samples from days 7/14/21. **A** The proportions of the different immune cell types are shown for the different incubation times. Significant p-values are highlighted with an asterisk (*: *p* < 0.05). The proportions of the different immune cell types are shown for the different incubation times (median along with the interquartile range). **B** Results are shown for the cases where corresponding 3D-OTC samples from days 7/14/21 could be analyzed. The immune cell composition shows significant longitudinal differences (***: *p* < 0.001). Prim: Primary tumor tissue, d7/d14/d21: 3D-OTC samples cultured for 7/14/21 days

Immunohistochemically stained slides were evaluated using QuPath after staining for CD3 (T lymphocytes), CD20 (B lymphocytes), CD56 (NK cells) and CD68 (macrophages) (Fig. 3A). We observed a significant decrease in the density of CD3-positive cells (*p* = 0.016/0.016/0.031) and of CD56-positive cells (*p* = 0.016/0.031/0.031) comparing primary tumor tissue with 3D-OTC samples (primary vs. day 7/primary vs. day 14/primary vs. day 21, Additional file 1: Table 5). For CD20-positive cells significant differences in the immune cell density could only be detected comparing primary tumor tissue with 3D-OTC samples cultured for 14 days (*p* = 0.016). The immune cell density for CD68-expressing cells was comparable between primary tumor tissue and 3D-OTC samples throughout the cultivation period.

No significant changes could be detected in the immune cell density of CD3-/CD20-/CD56-/CD68-expressing cells among the 3D-OTC samples during cultivation (day 7 vs. day 14, day 14 vs. day 21, day 7 vs. day 21).

Similarly, we could not detect significant differences in the expression of Ki67 in primary tumor tissue and the 3D-OTC samples cultivated for 7, 14 and 21 days (Additional file 1: Fig. S2C).

### Composition of the immune cell infiltrate

Our longitudinal analysis revealed significant differences in the composition of the immune cell infiltrate of primary tumor tissue compared to 3D-OTC samples cultured for 7, 14 and 21 days (*p* < 0.001, Fig. 3B and Additional file 1: Table 6). The most abundant cell type in primary tumor tissue and 3D-OTC samples were CD3-expressing T lymphocytes. The density of CD20-expressing B lymphocytes was decreased from day 7 to days 14/21. While the proportion of CD56-expressing NK cells was constant over the cultivation time, the density of CD68-expressing macrophages was increased in the 3D-OTC samples compared to primary tumor tissue.

### Gene expression in 3D-OTC samples and primary tumor tissue

Significantly more transcripts per 100 µm^2^ were detected in fresh frozen tissue compared to FFPE tissue (median fresh frozen tissue: 75, interquartile range 11, median FFPE tissue: 39, interquartile range 5, *p* = 0.029, Additional file 1: Fig. S5).

We detected significant differences for the centroids of the gene expression between all regions of interest within the 3D-OTC samples (non-invasive, invasive, expansive, central tumor areas, central stromal areas; Additional file 1: Table 7). Except for the comparison of invasive and central tumor regions of the 3D-OTC samples we could not detect significant differences in the dispersion of the gene expression between the groups.

We did not observe a significant difference for the centroids of the gene expression of invasive, non-invasive and expansive tumor areas of the 3D-OTC samples compared to the tumor periphery of primary tumor tissue. Furthermore, the centroids of the gene expression of central tumor parts of the 3D-OTC samples and central tumor areas of primary tumor tissue did not differ significantly as well as the centroids of the gene expression of central stromal areas of the 3D-OTC samples compared to peritumoral stroma of the primary tumor tissue. The dispersion of the gene expression differed significantly between non-invasive and expansive tumor areas of the 3D-OTC samples compared to primary tumor tissue. Similarly, the dispersion of the gene expression of central tumor areas of the 3D-OTC samples and central tumor areas of the primary tumor tissue differed significantly (Additional file 1: Table 7).

### Enriched gene sets in 3D-OTC regions of interest

We evaluated functional differences between all of the following regions of interest in gene set enrichment analysis using hallmark gene sets [19]: non-invasive tumor parts, invasive tumor parts, expansive tumor parts, central tumor parts and central stromal areas (Additional file 1: Fig. S6). Invasive tumor parts showed an up-regulation of genes involved in epithelial-mesenchymal transition, hypoxia, IL6 JAK STAT3 signaling, KRAS signaling, TNF-α signaling via NF-κB compared to central tumor areas and an up-regulation of genes involved in androgen response, apoptosis, E2F targets, estrogen response early, estrogen response late, G2M checkpoint, glycolysis, hypoxia, KRAS signaling dn, mitotic spindle, MYC targets, p53 pathway, peroxisome, PI3K AKT MTOR signaling, protein secretion, spermatogenesis, TNF-α signaling via NF-κB and UV response up compared to central stromal areas.

We could not detect a significant overlap between up-regulated and down-regulated genes and hallmark gene sets when we compared invasive tumor parts with non-invasive tumor parts and expansive tumor parts. Analyzing expansive tumor parts we could not detect an up-regulation or a down-regulation of genes compared to non-invasive tumor parts.

Analyzing central tumor parts we detected a down-regulation of genes involved in apical junction, epithelial mesenchymal transition, glycolysis and hypoxia in comparison to non-invasive tumor parts and a down-regulation of genes involved in apoptosis, E2F targets, G2M checkpoints, mitotic spindle, p53 pathway, pancreas beta cells, peroxisome, spermatogenesis and UV response up compared to expansive tumor parts. Central stromal parts showed a down-regulation of genes involved in apoptosis, E2F targets, estrogen response late, G2M checkpoint, glycolysis, hypoxia, IL6 JAK STAT3 signaling, mitotic spindle, MYC targets V1, p53 pathway, peroxisome, PI3K AKT MTOR signaling, protein secretion, spermatogenesis, UV response up compared to non-invasive tumor parts as well as an up-regulation of genes involved in angiogenesis, apical surface and IL6 JAK STAT3 signaling and a down-regulation of genes involved in E2F targets, estrogen response late, G2M checkpoint, glycolysis, mitotic spindle, p53 pathway, PI3K AKT MTOR signaling, protein secretion and spermatogenesis compared to expansive tumor parts. Central stromal areas showed an upregulation of genes involved in angiogenesis, apical surface, epithelial mesenchymal transition and IL6 JAK STAT3 signaling and a down-regulation of genes involved in apoptosis, E2F targets, estrogen response late, G2M checkpoint, glycolysis, mitotic spindle, MYC targets V1, p53 pathway, peroxisome, PI3K AKT MTOR signaling, protein secretion, spermatogenesis, UV response.

**Fig. 4:**
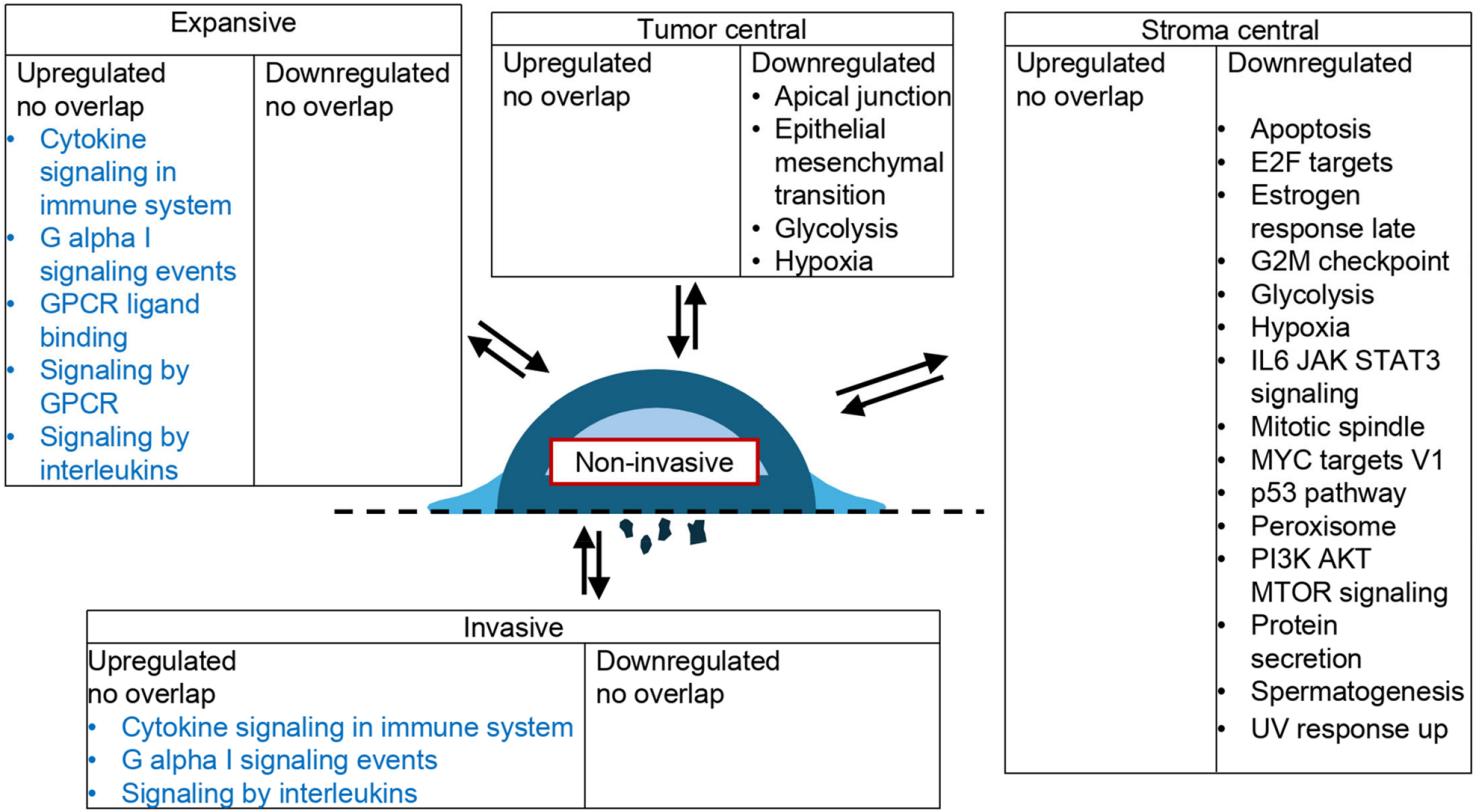
Functional differences between the investigated regions of interest. Up-regulated and down-regulated genes were included in gene set enrichment analysis and associations with hallmark and reactome gene sets were explored. Functional differences between invasive, expansive, central tumor and central stroma areas are shown with non-invasive tumor areas as reference. ● Hallmark gene sets,  Reactome gene sets

Figure 4 gives a detailed overview over the enriched hallmark gene sets in invasive, expansive, central tumor and central stroma areas with non-invasive tumor parts as respective references. Enriched hallmark gene sets with expansive, central tumor and central stroma areas are shown in Additional file 1: Fig. S7. Results for the transcriptomic analysis of each region of interest are shown in Additional file 2, the results of the gene set enrichment analysis using hallmark gene sets are shown in Additional file 3.

To better characterize invasive, non-invasive and expansive tumor areas beyond the hallmark gene sets we conducted gene set enrichment analysis utilizing the Reactome gene sets [22]. Here, we observed an up-regulation of genes involved in signaling by interleukins, cytokine signaling in immune system and G alpha I signaling events when we compared invasive and non-invasive tumor areas, an up-regulation of genes involved in signaling by interleukins, cytokine signaling in immune system, G alpha I signaling events, GPCR ligand binding and signaling by GPCR when we compared invasive and expansive tumor parts and an upregulation of genes involved in cytokine signaling in immune system, signaling by interleukins, G alpha I signaling events, GPCR ligand binding and signaling by GPCR when we compared expansive and non-invasive tumor parts (Fig. 4, Additional file 1: Fig. S7A, Additional file 4).

### Evaluation of potential drug responses

As a proof of concept we evaluated the translational implications of genes up-regulated between invasive and non-invasive tumor areas of the 3D-OTC samples and the response to established therapeutic medications in HNSCC. The following genes were up-regulated in invasive compared to non-invasive parts of the tumors: *ADAMTS1*,* CD83*,* CSF3*,* CXCL6*,* GEM*,* GNG11*,* IL1RL1*,* IL3RA*,* RND1*,* SNAI1* and *TENT5C*. We interrogated two web-based interfaces regarding the association between the up-regulated genes and the response to chemotherapeutics. The “HNCDrugResDb” indicated a resistance of invasive tumor parts to cetuximab, cisplatin and paclitaxel due to the up-regulation of *SNAI1*. No association could be detected with the remaining drugs under investigation (pingyangmycin, paclitaxel, trametinib, apatinib, 5-fluorouracil, dasatinib, bleomycin, panobinostat, vincristine, docetaxel, doxorubicin and gemcitabine). Similarly, “A database of genes related to platinum resistance” depicted an up-regulation of *SNAI1* in platinum-resistant cells due to an upregulation of cancer stem cells and epithelial mesenchymal transition pathways.

## Discussion

In translational research in vitro models could help to assess the efficacy of different therapeutic options and thus, improve personalized treatment regimens [8]. Ideally, the model systems should resemble primary tumor tissue and therefore, meet several requirements such as preserving the tissue architecture and cellular composition, and simulating the cancer ecosystem with limited nutrition and oxygenation [23]. In our study we aimed to further evaluate the 3D-OTC model previously described by Engelmann et al. in terms of its comparability to primary tumor tissue and its applicability in personalized cancer treatment [7].

We hypothesized that the growth patterns observed in 3D-OTC samples are associated with the aggressiveness of a tumor. Accordingly, we assumed that an invasive growth pattern at the DE would reflect a more aggressive tumor biology than the expansive or dormant growth patterns [7]. However, in HNSCC primary tumor tissue the morphologic assessment of tumor aggressiveness is hampered by the lack of a convincing prognostic grading system [24]. In many solid tumor entities including HNSCC tumor budding is the morphologic correlate of aggressive tumors and therefore, could be used for the prognostic stratification of the tumors [11, 25–28]. The expression of the proliferation marker Ki67 was previously also shown to be a prognostic biomarker in HNSCC with high Ki67 proliferation indices being associated with poor prognosis [29].

We observed a strong relationship between the number of tumor buds in the resection specimens and the number of tumor buds in the 3D-OTC models. Similarly, we saw an association between high tumor budding activity in the resection specimens and an invasive growth pattern at the DE of the 3D-OTC specimens supporting our hypothesis that the invasive growth pattern is associated with more aggressive tumor behavior. For other histomorphological risk factors like lymphovascular invasion and perineural invasion as well as the expression of the proliferation marker Ki67 we could not observe an association with the growth patterns of the 3D-OTC samples [30].

At the transcriptomic level aggressive tumors are thought to be associated with epithelial-mesenchymal transition, a process in which tumor cells lose their cell-cell adhesion and begin to invade the adjacent stroma [31, 32]. The results of our spatially resolved gene expression analysis further endorse this hypothesis: We observed an up-regulation of genes associated with epithelial-mesenchymal transition in the tumor areas that started to infiltrate the DE compared to central tumor parts [33]. Interestingly, we also detected an up-regulation of genes involved in the hallmark gene set “IL6 JAK STAT3 signaling”. For HNSCC it could be shown that an abnormal activation of the JAK/STAT signaling pathway leads to tumor progression and is also associated with immune suppression and degradation of the extracellular matrix [34].

Taken together, we believe the 3D-OTC model mimics the aggressive behavior of primary tumor tissue morphologically represented by an invasion of the DE.

Another advantage of the 3D-OTC model is the preservation of the immune tumor microenvironment that we observed in our longitudinal analysis. To determine the exact composition of the immune cell infiltrate we performed an immunohistochemical analysis of the 3D-OTC samples and compared the density of immune cells (T cells, B cells, NK cells and macrophages) and their composition to the primary tumor tissue.

Among immune cells, T lymphocytes seem to be particularly important for the efficacy of immune checkpoint inhibitors but monocytes also appear to play an important role [35, 36]. While we observed significantly lower amounts of T lymphocytes and NK cells, and to a lesser extent of B lymphocytes in the 3D-OTC models compared to primary tumor tissue, no changes in the macrophage density could be detected. Notably, the number of immune cells in the 3D-OTC models remained constant (on a relatively low level) throughout the cultivation period (days 7 to 21). On a transcriptomic level we observed an up-regulation of genes involved in signaling by interleukins and cytokine signaling in the immune system when we compared invasive tumor parts which infiltrated the DE and expansive tumor areas which grew above the DE. As chronic inflammation can lead to tissue damage and contribute to cancer onset and its advancement modulating the immune response could help to prevent tumor cells from invading the DE [37, 38]. Therefore, the 3D-OTC model might help to unravel the biological mechanisms which cause a more aggressive invasive phenotype compared to a more expansively proliferating phenotype.

These results may indicate that the 3D-OTC model could especially be used to test the response of tumors to immune checkpoint inhibitors and in particular in a contemporary neoadjuvant or adjuvant setting (Keynote-689 protocol, NIVOPOSTOP protocol) [39, 40]. However, in our study we only analyzed the immune cell infiltrate of the 3D-OTC samples and did not administer immune checkpoint inhibitors to the 3D-OTC samples. Furthermore, it is still unclear whether the decrease in immune cell numbers hinders the applicability of the 3D-OTC model to predict therapeutic response.

We also believe that one further advantage of the 3D-OTC model is the cultivation on top of a DE which allows a standardized orientation of the tumor tissue and thus facilitates the microscopic determination of defined regions of interest (invasive/non-invasive/expansive/central tumor and stromal areas) e.g., for transcriptomic analysis. The use of spatially resolved gene expression analysis helps to detect changes in the gene expression profiles of aggressive tumors and therefore, can help to determine the therapy response more precisely and at the gene expression level [41].

We could not detect significant differences for the centroids and dispersion of the gene expression between the regions of interest of the 3D-OTC samples (invasive, non-invasive and expansive tumor areas of the 3D-OTC samples and tumor periphery of the primary tumor tissue; central tumor areas of the 3D-OTC samples and central tumor areas of the primary tumor tissue; central stromal areas of the 3D-OTC samples and peritumoral stroma of the primary tumor tissue) indicating that the regions of interest in the 3D-OTC samples can simulate the conditions of primary tumor tissue in principle. However, the cohort size of our study was rather small, which limits the generalizability of our results and the pre-designed panel used for the “Xenium in situ” covers only the transcriptome of *n* = 377 genes.

Functionally, we observed differentially expressed genes between the regions of interest in the 3D-OTC samples which in principle enables the assessment of therapy resistance and monitoring of targeted therapies in vitro. As a proof of concept, we compared invasive and non-invasive parts of the 3D-OTC specimens and identified *SNAI1* as an up-regulated gene which could lead to resistance to cisplatin- and cetuximab-based chemotherapy and therefore, could be associated with poor outcomes of patients [42–44]. Cisplatin and cetuximab comprise two drugs which are regularly administered to head and neck cancer patients (EXTREME regimen, TPEx regimen). Therefore, in vitro drug testing assays applying the 3D-OTC model could help to identify resistant tumor subclones leading to the replacement of cisplatin/cetuximab by presumably more effective substances [45]. Thus, we believe the standardized evaluation of different regions of interest in the 3D-OTC samples could help to choose more effective chemotherapeutics and could help to optimize the treatment of HNSCC patients.

However, the functional and predictive significance of our proposed regions of interest have to be evaluated in further studies analyzing larger series of 3D-OTC samples. Furthermore, in our study only male patients were included, limiting the generalization of our results. A further limitation of cancer in vitro models is the fact that the models might not capture the whole intra-tumor heterogeneity and/or some tumor subclones might be lost during the cultivation process which could explain therapy resistance of tumor subclones in drug testing assays [46, 47]. Nevertheless, it has been shown that other in vitro models like organoids can sustain key genetic alterations and subclones of their primary tumor tissue [48]. Regarding tumor heterogeneity we observed significant differences in the variance of tumor buds (but not the immune cell infiltrate) in the 3D-OTC samples. In order to capture the intra-tumor heterogeneity as well as possible the tissue segments dissected from the main tumor and subsequently cultured as 3D-OTC samples should be as large as possible. Although we have successfully cultured a mean of *n* = 9 3D-OTC samples per case to cover different tumor areas the model is limited in representing tumor heterogeneity and generalizability especially regarding the heterogeneity of the immune microenvironment [49]. Moreover, the cultivation of 3D-OTC samples is complex, and not all 3D-OTC samples can be cultured (for technical reasons; according to our experience > 80% successful cultivations) as some 3D-OTC models were mainly necrotic. The significant loss of immune cells during the cultivation period might also limit the model’s applicability in immunotherapy studies. The observed reduction in T-cells, NK-cells, and B-cells could be attributable to the lack of exogenous lymphocyte-supporting cytokines under the current culture conditions and systematic optimization and validation of these conditions should be evaluated in future studies. Importantly, monocytes demonstrate robust survival and stability within the system over extended culture periods which makes the model particularly well suited for studies investigating monocyte functions. The pre-designed Xenium in situ panel covered *n* = 377 genes but several genes which might be of therapeutic interest (e.g., PI3K because of potential PI3K inhibitors) and GSK3B which might be used as a biomarker for metastatic spread were not included in the panel limiting the informative value of the targeted panel [50, 51].

## Conclusions

We evaluated the 3D-OTC model for its general applicability as an in vitro platform to simulate primary tumor tissue. The growth pattern of cancer cells on the dermal equivalent is associated with an aggressive tumor phenotype. While the immune cell infiltrate of the 3D-OTC samples was significantly lower for certain immune cells compared to primary tumor tissue, the immune cell density remained constant over the time of cultivation (days 7 to 21). Spatially resolved gene expression analysis of defined regions of interest might help to allow for various tests used in personalized medicine and drug screening for patients. Therefore, we believe the 3D-OTC model may be suitable to test for the response of HNSCC to therapeutic interventions in vitro.

## Supplementary Information

Below is the link to the electronic supplementary material.

Supplementary Material 1

Supplementary Material 2

Supplementary Material 3

Supplementary Material 4

## Data Availability

Spatial transcriptomic data is available at the Gene Expression Omnibus (https://www.ncbi.nlm.nih.gov/geo/) under the accession number GSE324934.

## Abbreviations

3D: OTC three-dimensional organotypic co-culture
DE: dermal equivalent
FFPE: formalin-fixed paraffin-embedded
H&E: hematoxylin and eosin
HNSCC: head and neck squamous cell carcinoma
HPF: high-power field
IHC: immunohistochemistry
