# Supplementary material for: Personalizing the treatment of head and neck cancer in vitro: The 3D-OTC model
Source: BMC Med. 2026 Apr 25;24:270. doi: 10.1186/s12916-026-04883-z (PMC13123083; doi:10.1186/s12916-026-04883-z)
Supplement: Supplementary file 1 — Supplementary Material 1 [file 12916_2026_4883_MOESM1_ESM.docx]

**Additional file 1: Table 1:** Clinicopathological characteristics of the patient cohort.

| **OTC number** | **Age** | **Sex** | **Localization primary tumor** | **p16** | **pT** | **pN** | **Extracapsular extension** | **Lymphovascular invasion** | **Angioinvasion** | **Perineural invasion** | **Grading** | **R-Status** | **Morphology** | **Recurrence** | **Overall survival** |
| --- | --- | --- | --- | --- | --- | --- | --- | --- | --- | --- | --- | --- | --- | --- | --- |
| 1 | 37 | male | Larynx |  | 4 | 0 | no | absent | present | present | 2 | 0 | keratinizing | no | alive |
| 2 | 70 | male | Oral cavity |  | 2 | X | - | absent | absent | absent | 3 | 0 | keratinizing | yes | alive |
| 3 | 67 | male | Larynx |  | 1 | 0 | no | absent | absent | absent | 2 | 0 | non-  keratinizing | no | alive |
| 4 | 73 | male | Larynx |  | 4 | 1 | no | present | absent | present | 3 | 0 | keratinizing | no | alive |
| 5 | 54 | male | Larynx |  | 2 | 1 | no | present | absent | present | 2 | 0 | non-  keratinizing | no | dead |
| 6 | 83 | male | Larynx |  | 4 | 0 | no | absent | absent | present | 2 | 0 | keratinizing | no | alive |
| 7 | 50 | male | Oropharynx | negative | 2 | 3 | yes | present | absent | absent | 2 | X | keratinizing | no | dead |
| 8 | 66 | male | Larynx |  | 4 | 3 | yes | present | absent | absent | 2 | 0 | keratinizing | no | alive |
| 9 | 58 | male | Oropharynx | positive | 2 | 2 | yes | present | absent | absent | 2 | X | keratinizing | no | alive |
| 10 | 57 | male | Oropharynx | negative | 1 | 3 | yes | absent | absent | absent | 2 | X | keratinizing | no | alive |

**Additional file 1: Table 2:** Parameters used for the “Positive cell detection” algorithm in QuPath.

|  | **CD3** | **CD20** | **CD56** | **CD68** |
| --- | --- | --- | --- | --- |
| Detection image | Hematoxylin OD | Hematoxylin OD | Hematoxylin OD | Hematoxylin OD |
| Requested pixel size | 0.5 µm | 0.5 µm | 0.5 µm | 0.5 µm |
| Background radius | 0 µm | 0 µm | 0 µm | 0 µm |
| Use opening by reconstruction | on | on | on | on |
| Median filter radius | 0 µm | 0 µm | 0 µm | 0 µm |
| Sigma | 1.0 µm | 1.0 µm | 1.0 µm | 1.0 µm |
| Minimum area | 10 µm2 | 10 µm2 | 10 µm2 | 10 µm2 |
| Maximum area | 50 µm2 | 50 µm2 | 50 µm2 | 50 µm2 |
| Threshold | 0.1 | 0.1 | 0.1 | 0.1 |
| Max background intensity | 2 | 2 | 2 | 2 |
| Split by shape | on | on | on | on |
| Exclude DAB (membrane staining) | off | off | off | off |
| Cell expansion | 1.5 µm | 1.5 µm | 1.5 µm | 1.5 µm |
| Include cell nucleus | on | on | on | on |
| Smooth boundaries | on | on | on | on |
| Make measurements | on | on | on | on |
| Score compartment | Nucleus: DAB OD mean | Nucleus: DAB OD mean | Nucleus: DAB OD mean | Nucleus: DAB OD mean |
| Threshold 1+ | 0.1 | 0.2 | 0.2 | 0.3 |
| Threschold 2+ | 0.4 | 0.4 | 0.4 | 0.4 |
| Threschold 3+ | 0.6 | 0.6 | 0.6 | 0.6 |
| Single threshold | on | on | on | on |

**Additional file 1: Table 3:** Gene panel applied in the “Xenium in situ” analysis.

| ABCC11 | CAV1 | CLECL1 | FCER1A | IL1RL1 | MS4A1 | RGS16 | TFF2 |
| --- | --- | --- | --- | --- | --- | --- | --- |
| ACE2 | CAVIN1 | CLIC6 | FCGR1A | IL2RA | MS4A2 | RIDA | TFPI |
| ACKR1 | CAVIN2 | CNN1 | FCGR3A | IL3RA | MS4A4A | RND1 | THAP2 |
| ACTA2 | CCDC39 | COCH | FCN1 | IL7R | MS4A6A | RTKN2 | THBS2 |
| ACTG2 | CCDC78 | COL17A1 | FCN2 | INMT | MTRNR2L11 | S100A1 | THY1 |
| ADAM28 | CCL19 | COL5A2 | FGFBP1 | INS | MYBPC1 | S100A12 | TIMP4 |
| ADAMTS1 | CCL27 | CPA3 | FGFBP2 | IRF8 | MYC | SCGB2A1 | TM4SF18 |
| ADGRE1 | CCL5 | CRHBP | FGL2 | KCNK3 | MYH11 | SCGN | TM4SF4 |
| ADGRL4 | CCNB2 | CRISPLD2 | FHL2 | KCNMA1 | MYLK | SELE | TMC5 |
| ADH1C | CCR2 | CSF2RA | FKBP11 | KIT | MZB1 | SELL | TMEM100 |
| ADH4 | CCR7 | CSF3 | FOXA1 | KLK11 | NAT8 | SEMA3C | TMEM174 |
| ADIPOQ | CD14 | CTLA4 | FOXI1 | KLRB1 | NKG7 | SERPINB2 | TMEM52B |
| AGER | CD163 | CTSG | FOXJ1 | KLRC1 | NPDC1 | SERPINB3 | TNC |
| AGR3 | CD19 | CTSK | FOXP3 | KLRD1 | NTN4 | SERPINB9 | TNFRSF13B |
| AHSP | CD1A | CXCL10 | FSTL3 | KNG1 | OGN | SFRP2 | TNFRSF17 |
| AIF1 | CD1C | CXCL2 | FXYD2 | KRT20 | OPRPN | SFRP4 | TNFRSF9 |
| ALAS2 | CD1E | CXCL6 | GATA2 | KRT7 | PCNA | SFTA2 | TOP2A |
| ALDH1A3 | CD2 | CXCL9 | GATM | LAG3 | PCOLCE | SH2D3C | TRAC |
| AMY2A | CD247 | CXCR4 | GCG | LAMP3 | PCP4 | SLAMF1 | TREM2 |
| ANGPT2 | CD27 | CYP1A1 | GDF15 | LGI4 | PCSK2 | SLAMF7 | TSPAN19 |
| ANPEP | CD274 | CYP2A7 | GEM | LGR5 | PDCD1 | SLC18A2 | UBE2C |
| APCDD1 | CD28 | CYP2B6 | GHRL | LIF | PDGFRA | SLC22A8 | UMOD |
| APOA5 | CD300E | CYP2F1 | GKN2 | LILRA4 | PDGFRB | SLC26A2 | UPK3B |
| APOBEC3A | CD34 | CYP3A4 | GLIPR1 | LILRA5 | PDPN | SLC26A3 | VCAN |
| APOLD1 | CD3D | CYP4B1 | GLYATL1 | LILRB2 | PEBP4 | SLC4A1 | VSIG4 |
| AQP2 | CD3E | CYTIP | GNG11 | LILRB4 | PECAM1 | SMIM24 | VWA5A |
| AQP3 | CD4 | DERL3 | GNLY | LPL | PGR | SMYD2 | VWF |
| AQP8 | CD5L | DES | GPC1 | LTBP2 | PLA2G7 | SNAI1 |  |
| AQP9 | CD68 | DIRAS3 | GPC3 | LY6D | PLAC9 | SNCA |  |
| AR | CD69 | DMBT1 | GPR183 | LY86 | PLCG2 | SNCG |  |
| ARFGEF3 | CD70 | DNAAF1 | GPRC5A | LYVE1 | PLD4 | SNTN |  |
| ASCL1 | CD79A | DNASE1L3 | GPX2 | MALL | PLIN4 | SOX17 |  |
| ASCL3 | CD83 | DPEP1 | GYPA | MAMDC2 | PMP22 | SOX18 |  |
| ASPN | CD86 | DPT | GYPB | MARCO | PPARG | SOX2 |  |
| BAMBI | CD8A | DST | GZMA | MCEMP1 | PPP1R1A | SPDEF |  |
| BANK1 | CD93 | DUSP2 | GZMB | MCF2L | PPP1R1B | SPI1 |  |
| BASP1 | CDH16 | ECSCR | GZMK | MDM2 | PPY | SPIB |  |
| BBOX1 | CDK1 | EDN1 | HAMP | MEDAG | PRDM1 | SRPX |  |
| BCL2L11 | CENPF | EDNRB | HAVCR2 | MEF2C | PRF1 | SST |  |
| BMX | CFAP53 | EGFL7 | HEMGN | MEST | PRG4 | STC1 |  |
| BTNL9 | CFB | EGFR | HEPACAM2 | MET | PROX1 | STC2 |  |
| C15orf48 | CFHR1 | EHF | HES4 | MFAP5 | PTGDS | STEAP4 |  |
| C1orf162 | CFHR3 | ELF5 | HIGD1B | MKI67 | PTN | TAC1 |  |
| C1orf194 | CFTR | EPCAM | HLA-DQB2 | MLANA | PTPRC | TAT |  |
| C20orf85 | CHGA | ERBB2 | HMGCS2 | MLPH | PVALB | TBX3 |  |
| C5orf46 | CLCA1 | ERG | HPGDS | MMRN1 | RAMP2 | TCF15 |  |
| C6orf118 | CLCA2 | ESR1 | HPX | MMRN2 | RAPGEF3 | TCF4 |  |
| C7 | CLEC10A | FAS | IGF1 | MNDA | RBP5 | TCIM |  |
| CA4 | CLEC14A | FBLN1 | IGSF6 | MPEG1 | RERGL | TCL1A |  |
| CAPN8 | CLEC4E | FBN1 | IL1R2 | MRC1 | RETN | TENT5C |  |

**Additional file 1: Table 4:** R packages used for statistical analysis and visualization.

| apeglm (1.28.0) | AnnotationDbi (1.68.0) | ashr (2.2-67) | Biobase (2.66.0) | BiocGenerics (0.52.0) |
| --- | --- | --- | --- | --- |
| BioParallel (1.40.2) | clusterProfiler (4.14.6) | DESeq2 (1.46.0) | dplyr (1.1.4) | enrichplot (1.26.6) |
| fgsea (1.32.4) | forcats (1.0.0) | genefilter (1.88.0), | GenomeInfoDb (1.42.3) | ggplot2 (4.0.1) |
| ggrepel (0.9.6) | GenomicRanges (1.58.0) | IRanges (2.40.1) | lubridate (1.9.4) | Matrix (1.7-4) |
| MatrixGenerics (1.18.1) | matrixStats (1.5.0) | mgcv (1.9.3) | msigdbr (24.1.0) | nlme (3.1-168) |
| org.Hs.eg.db (3.20.0) | permute (0.9-8) | purrr (1.0.4) | readr (2.1.5) | reshape2 (1.4.4) |
| S4Vectors (0.44.0) | stringr (1.5.1) | SummarizedExperiment (1.36.0) | sva (3.54.0) | tibble (3.2.1) |
| tidyr (1.3.1) | tidyverse (2.0.0) | vegan (2.7-2) |  |  |

**Additional file 1: Table 5:** Changes in the density of the different immune cell types over the cultivation period. Shown are the median and the interquartile range for the number of the corresponding immune cells per µm^2^.

| **Resection specimen** | **3D-OTC model day 7** | **3D-OTC model day 14** | **3D-OTC model 21** | **Immune cell** |
| --- | --- | --- | --- | --- |
| 1295 (1179) | 84 (80) | 105 (122.5) | 78.5 (38.75) | T lymphocyte (CD3) |
| 139 (118) | 6 (86) | 0 (0.5) | 1 (2.75) | B lymphocyte (CD20) |
| 262 (463) | 14 (15.5) | 9 (42.5) | 15 (8.25) | NK cell (CD56) |
| 20 (45) | 37 (47.5) | 18 (64) | 1 (44) | macrophage (CD68) |

**Additional file 1: Table 6:** Proportion of the different immune cell types with changes of the immune cell composition over the cultivation period. Shown are the median and the 95% confidence interval.

| **Resection specimen** | **3D-OTC model day 7** | **3D-OTC model day 14** | **3D-OTC model 21** | **Immune cell** |
| --- | --- | --- | --- | --- |
| 76.1%  (56.3%-86.9%) | 42.2%  (21.4%-63.1%) | 58.1%  (54.6%-75.3%) | 54.9%  (39.6%-72.4%) | T lymphocyte (CD3) |
| 8.4%  (7.4%-12.9%) | 35.0%  (3.2%-50.9%) | 0.5%  (0%-0.1%) | 1.4%  (0%-4.0%) | B lymphocyte (CD20) |
| 14.3%  (4.9%-29.3%) | 7.8%  (0.4%-9.5%) | 16.3%  (0%-21.8%) | 17.4%  (12.3%-19.6%) | NK cell (CD56) |
| 1.3%  (0.9%-1.9%) | 15.0%  (13.6%-31.3%) | 25.1%  (8.5%-28.2%) | 26.4%  (0%-45.9%) | macrophage (CD68) |

**Additional file 1: Table 7:** Results for the PERMANOVA test calculated for the regions of interest within the 3D-OTC model and for the regions of interest of the 3D-OTC model and the regions of interest in primary tumor tissue.

| **Within OTC** | **PERMANOVA test** | | **dispersion test (permutated)** | |
| --- | --- | --- | --- | --- |
| Regions of interest | R^2^ | p-value | F | p-value |
| invasive vs. non-invasive | 0.267 | 0.002 | 2.849 | 0.085 |
| invasive vs. expansive | 0.323 | < 0.001 | 4.020 | 0.078 |
| invasive vs. central tumor | 0.253 | < 0.001 | 8.318 | 0.022 |
| invasive vs. central stroma | 0.311 | 0.009 | 1.113 | 0.319 |
| non-invasive vs. expansive | 0.405 | < 0.001 | 0.007 | 0.911 |
| non-invasive vs. central tumor | 0.363 | < 0.001 | 0.581 | 0.464 |
| non-invasive vs. central stroma | 0.218 | < 0.001 | 0.006 | 0.975 |
| expansive vs. central tumor | 0.436 | < 0.001 | 0.630 | 0.450 |
| expansive vs. central stroma | 0.236 | < 0.001 | 0.020 | 0.899 |
| central tumor vs. central stroma | 0.298 | < 0.001 | 0.367 | 0.558 |
| **OTC vs. primary tumor tissue** | **PERMANOVA test (paired)** | | **dispersion test (permutated, paired)** | |
| invasive (OTC) vs. tumor periphery (primary) | 1 | > 0.999 | 7.268 | 0.5 |
| non-invasive (OTC) vs. tumor periphery (primary) | 1.621 | 0.333 | 3.036 | 0.042 |
| expansive (OTC) vs. tumor periphery (primary) | 2.410 | 0.167 | 4.571 | 0.042 |
| central tumor (OTC) vs. central tumor (primary) | 5.142 | 0.167 | 6.168 | 0.042 |
| central stroma (OTC) vs. peritumoral stroma (primary) | 0.076 | > 0.999 | 2.280 | 0.250 |


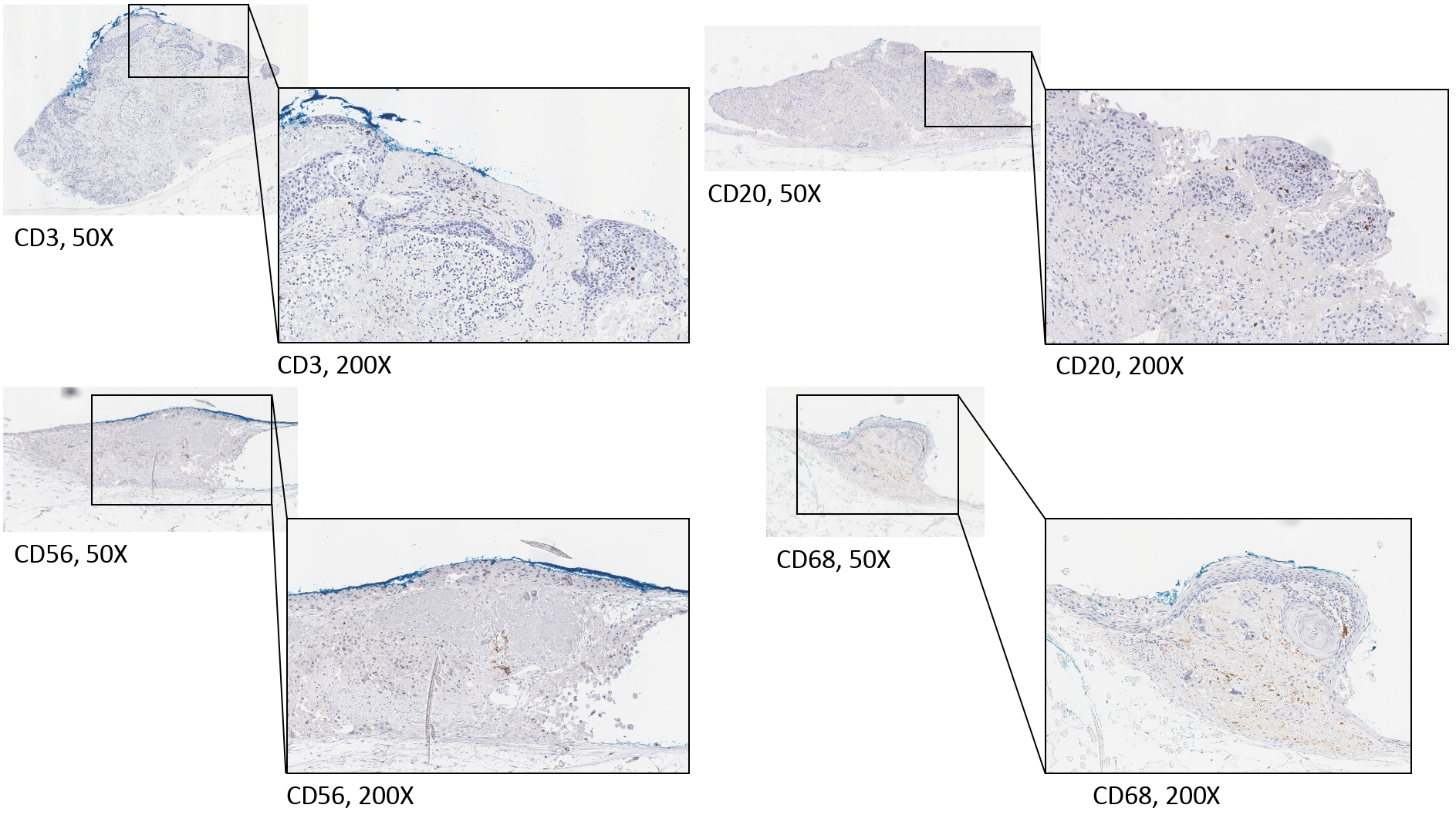


**Additional file 1: Fig. S1:** Examples of immunohistochemically stained 3D-OTC samples for CD3 (top left), CD20 (top right), CD56 (bottom left) and CD68 (bottom right).


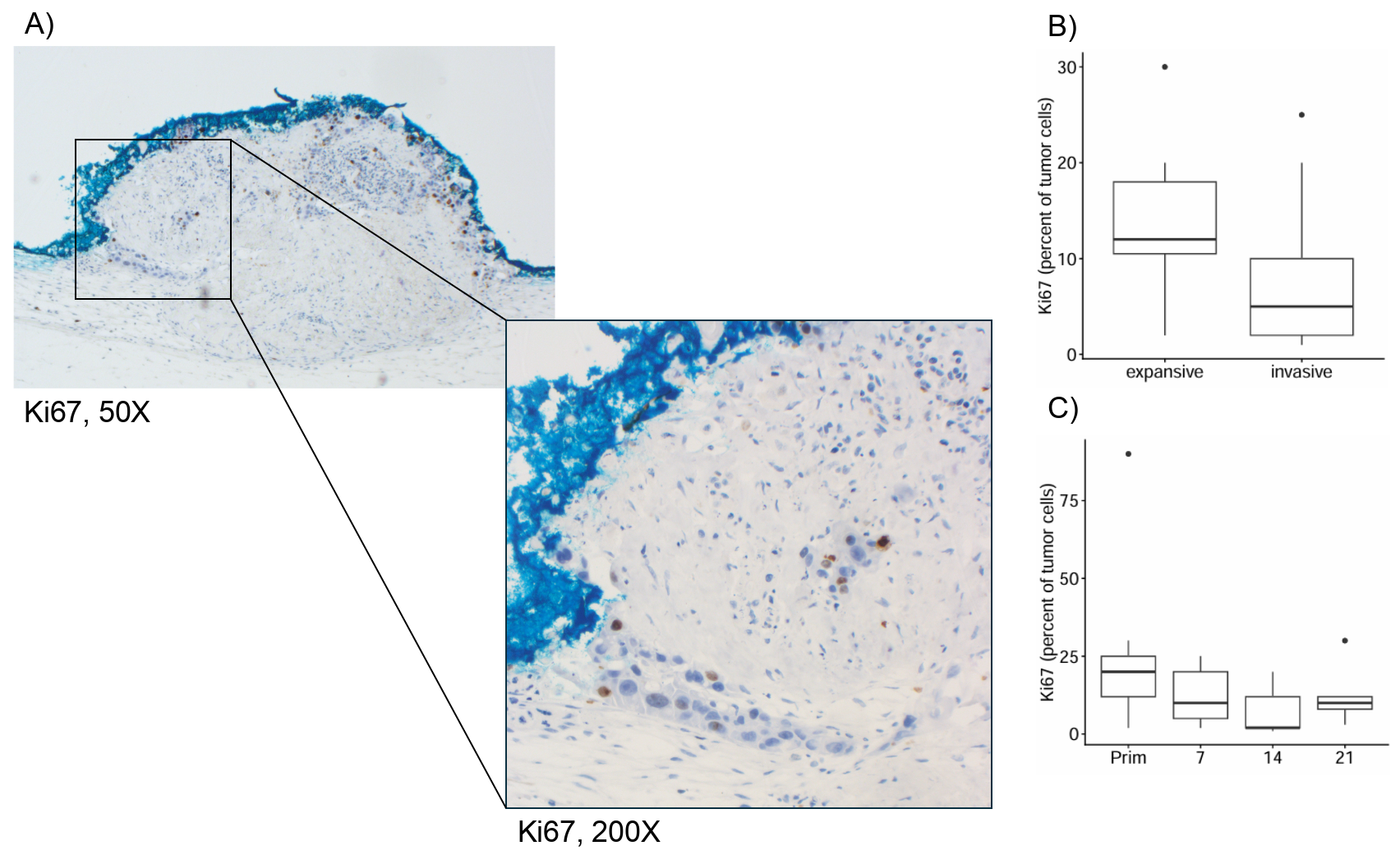


**Additional file 1: Fig. S2:** Example of an immunohistochemically stained 3D-OTC sample for Ki67 (A). The illustration was derived from 3D-OTC sample 8 cultivated for 14 days. No significant differences could be observed for the expression of the proliferation marker Ki67 when comparing the growth pattern at the DE with the proliferation of 3D-OTC samples (B). Similarly, no significant differences were observed between the proliferation of primary tumor tissue and 3D-OTC samples (C).


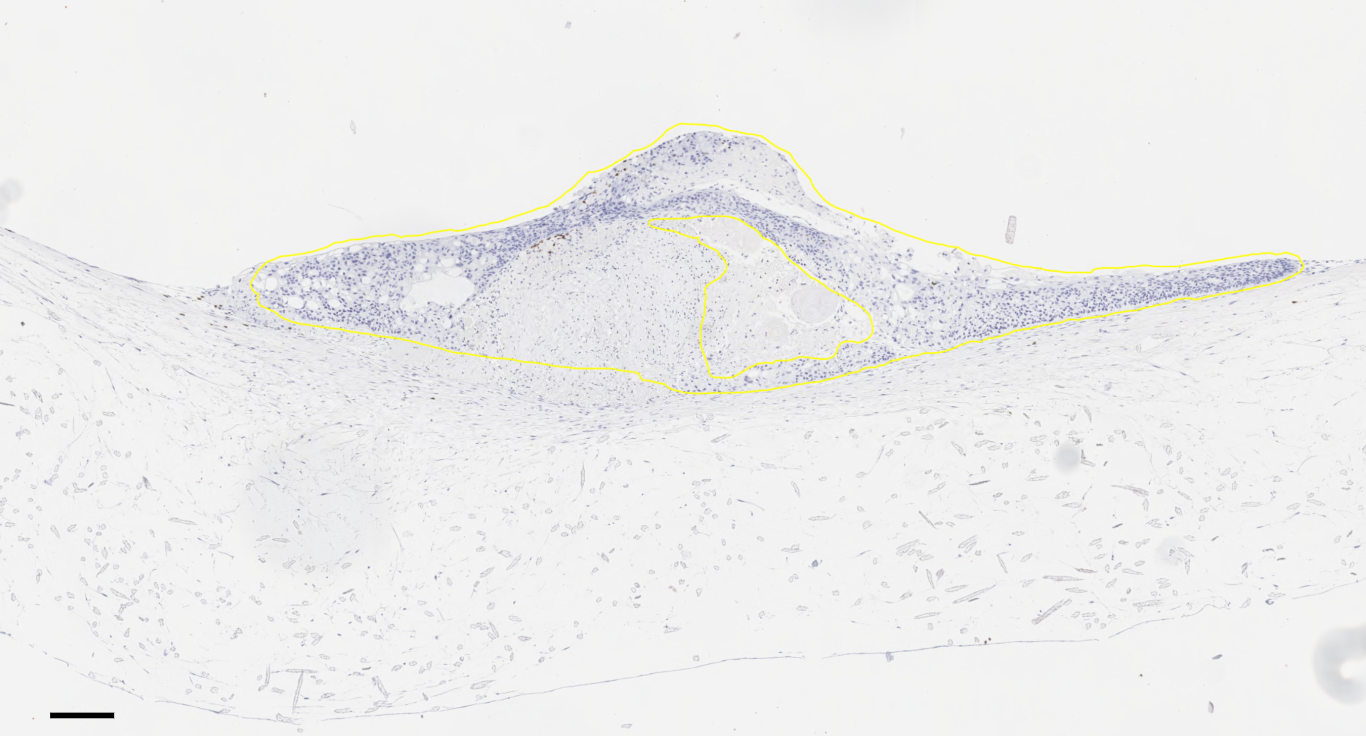


**Additional file 1: Fig. S3:** Annotation of the tumor outlines for the automated cell detection algorithm (CD3 immunohistochemistry). Scale bar 200 µm.


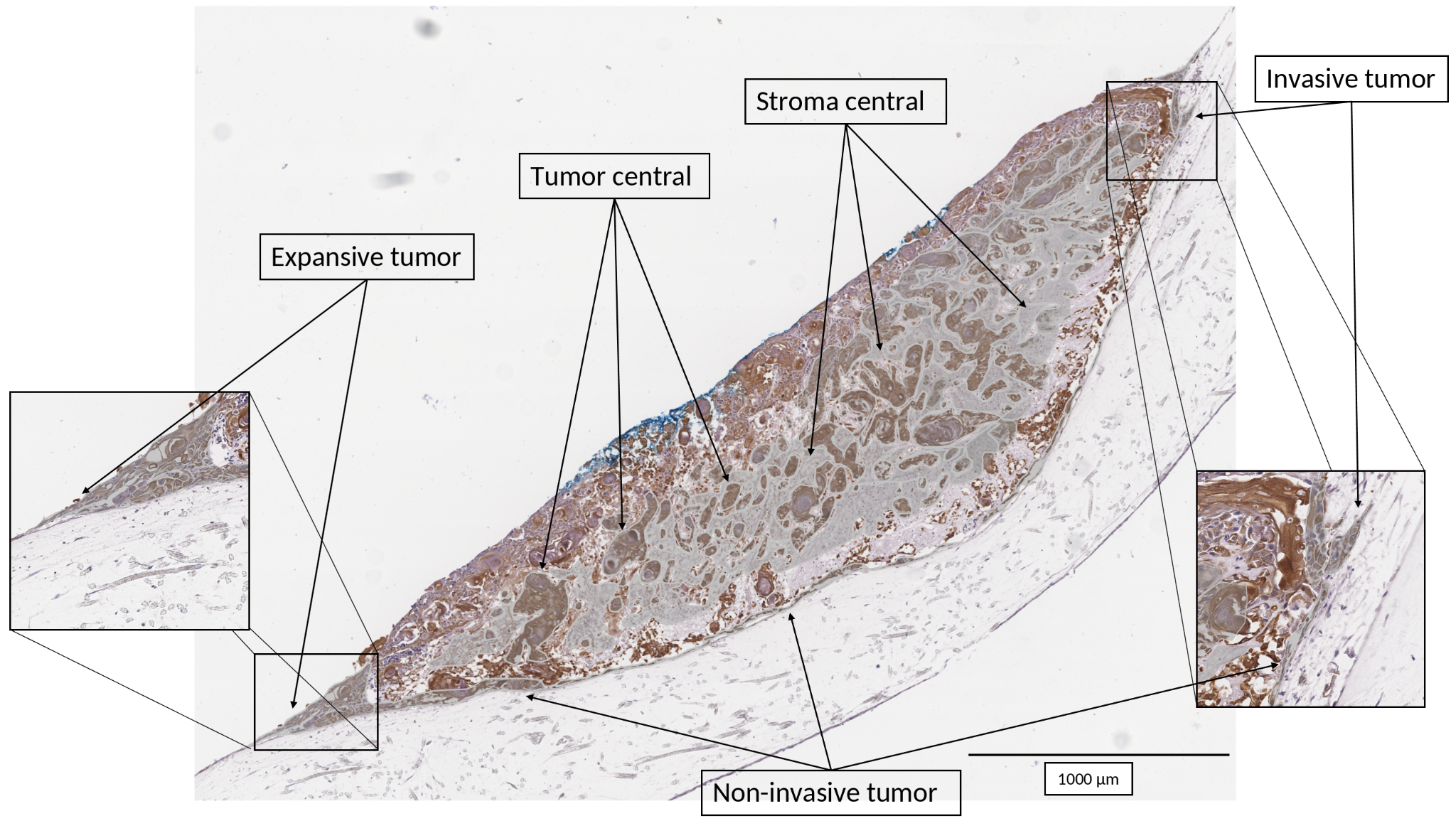


**Additional file 1: Fig. S4:** Example of a “Xenium in Situ” analysis using Xenium explorer. A DAPI stained section and an immunohistochemically stained section (CKpan) were coregistered. Tumor cells and stromal areas were then annotated on the immunohistochemically stained section and transcripts per µm^2^ were recorded. Necrotic areas were excluded from the analysis. The following regions of interest were analyzed: invasive tumor, non-invasive tumor, expansive tumor, central tumor areas and central stromal areas (CKpan immunohistochemistry, scalebar 1000 µm).


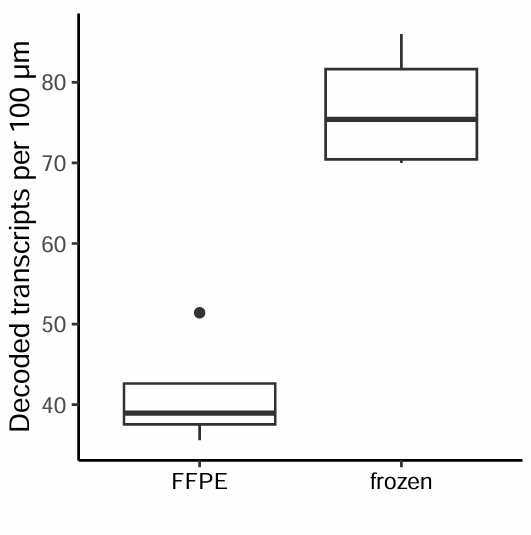


**Additional file 1: Fig. S5:** Comparison of decoded transcripts per 100 µm between FFPE tissue and fresh-frozen tissue. In fresh-frozen tissue significantly higher numbers of transcripts per 100 µm were detected.


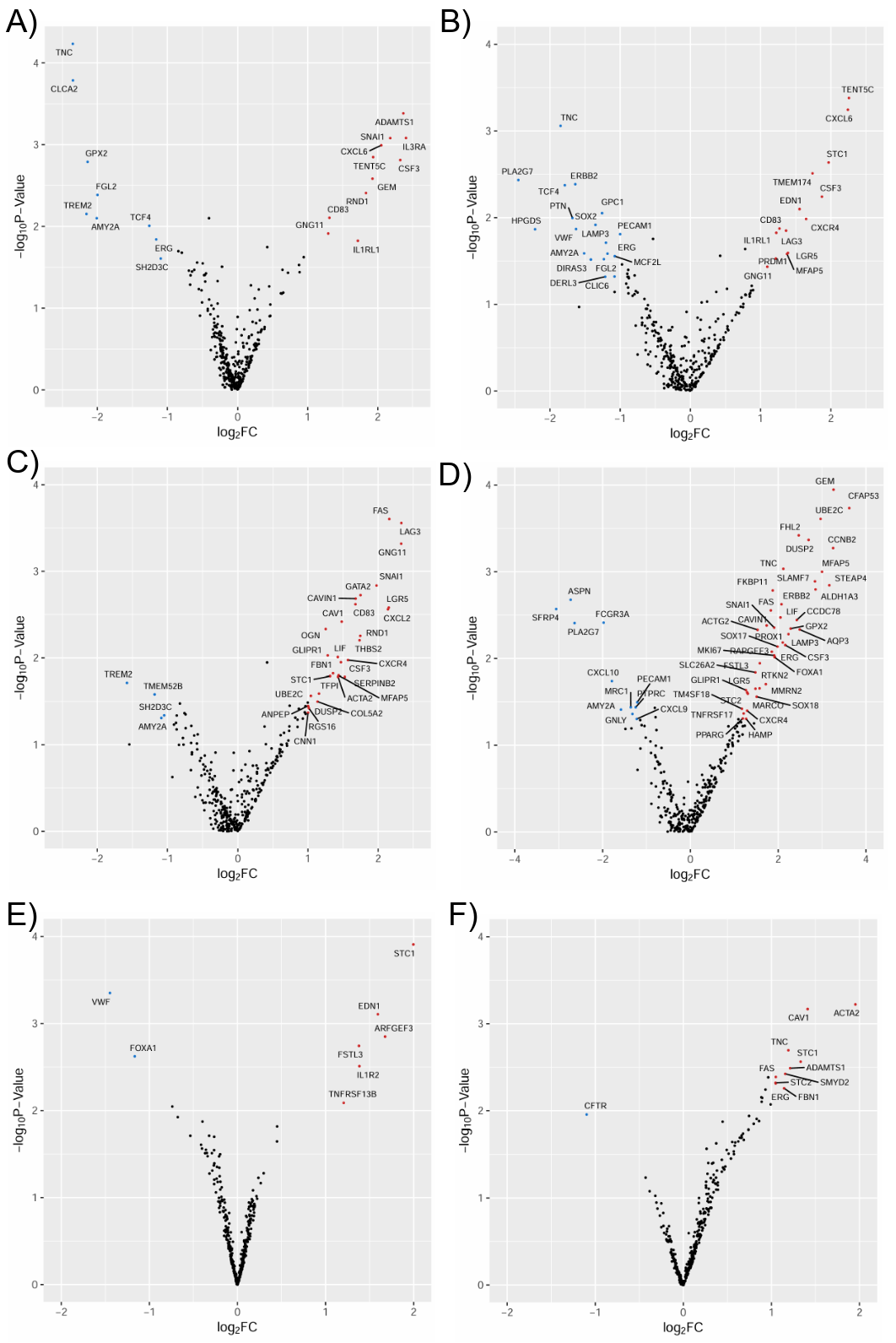


**Additional file 1: Fig. S6** (continued on next page).


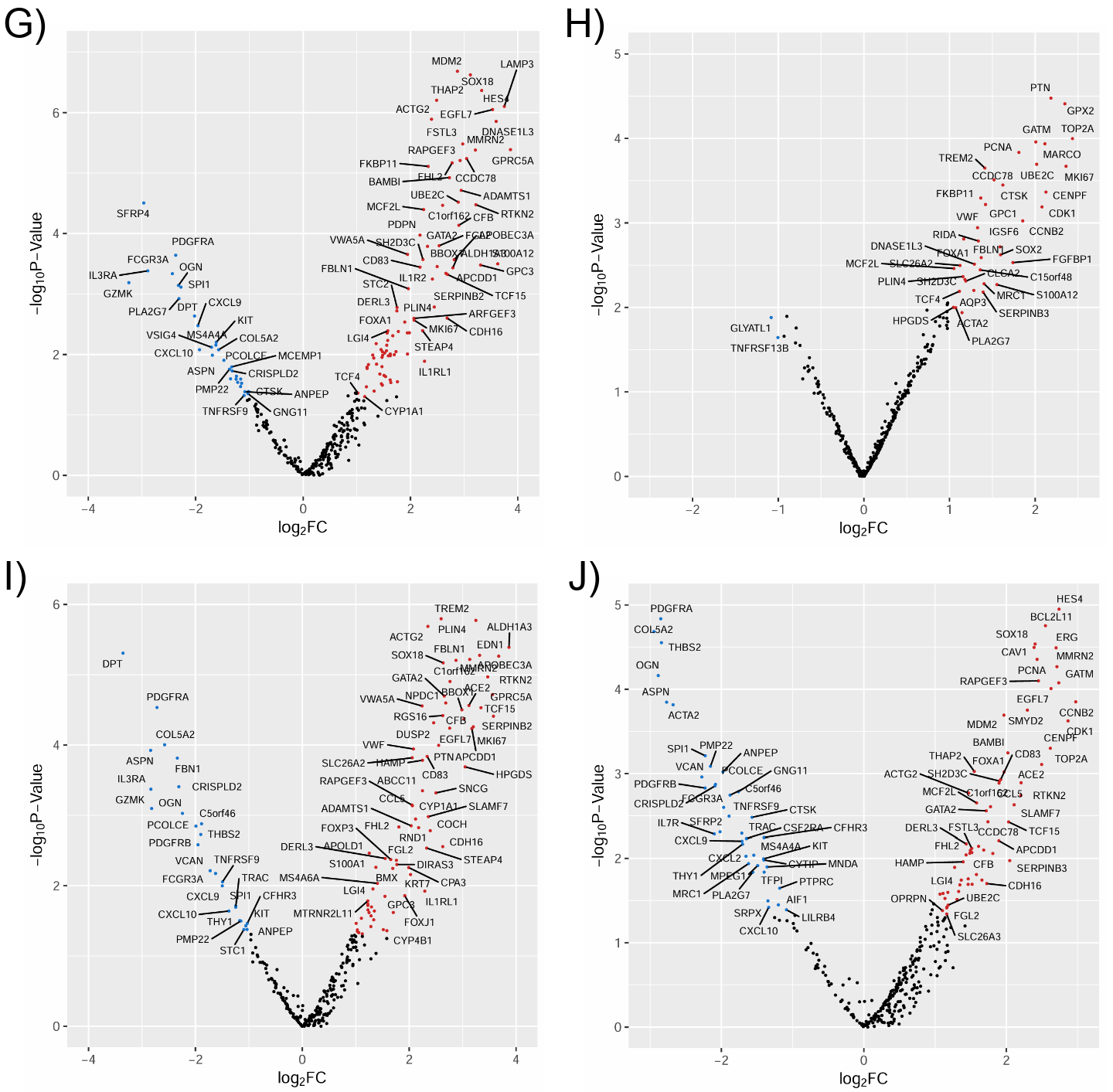


**Additional file 1: Fig. S6:** Volcano plots for differentially expressed genes between all regions of interest. Compared are invasive and non-invasive (reference) tumor parts (A), invasive and expansive (reference) tumor parts (B), invasive tumor parts and central tumor (reference) areas (C), invasive tumor parts and central stromal (reference) areas (D), non-invasive and expansive (reference) tumor parts (E), non-invasive tumor parts and central tumor (reference) areas (F), non-invasive tumor parts and central stromal (reference) areas (G), expansive tumor parts and central tumor (reference) areas (H), expansive tumor parts and central stromal (reference) areas (I) and central tumor parts and central stromal (reference) areas (J). Up-regulated genes are highlighted in red, down-regulated genes in blue.


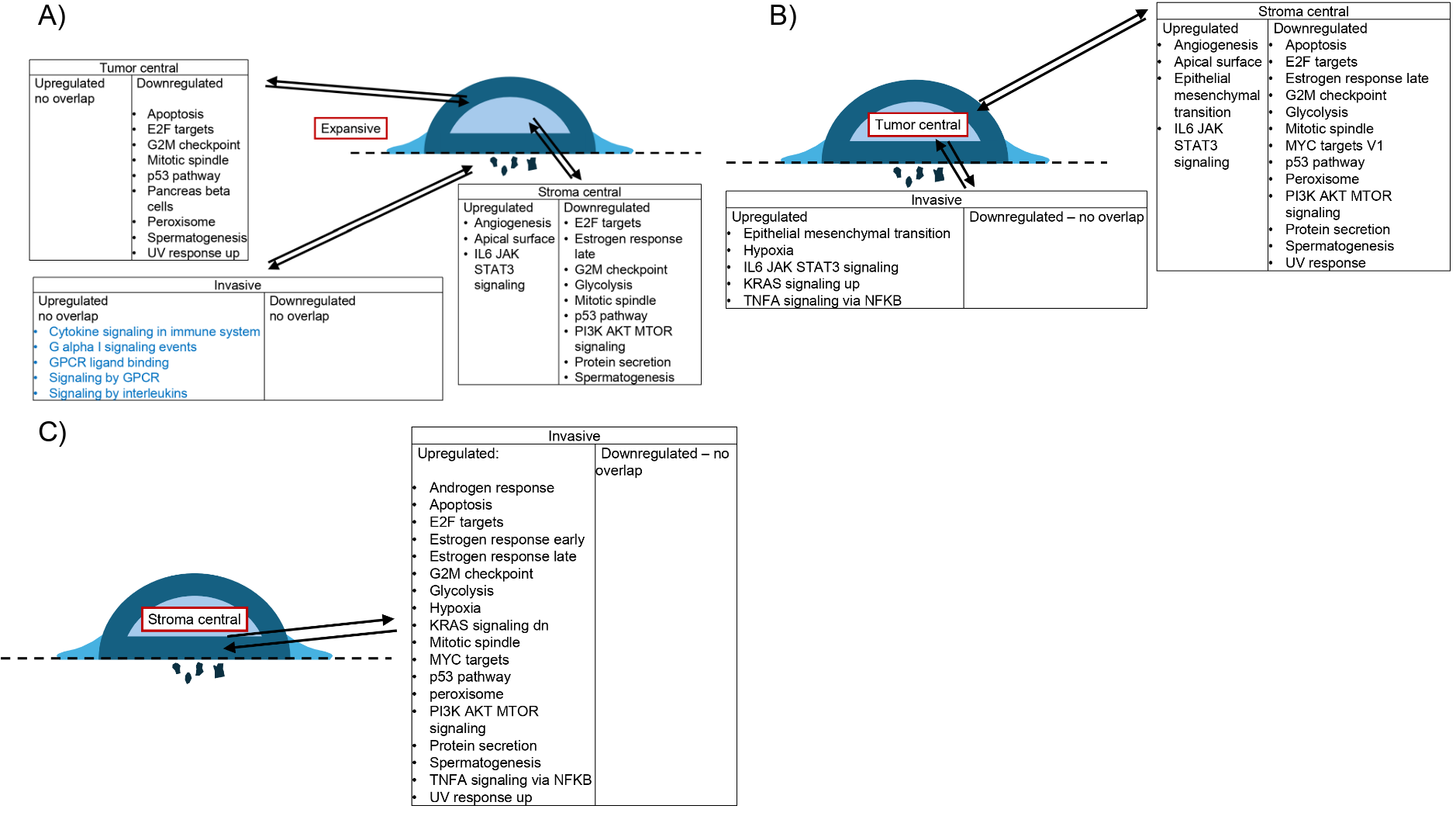


**Additional file 1: Fig. S7:** Functional differences between the investigated regions of interest. Up-regulated and down-regulated genes were included in gene set enrichment analysis and associations with hallmark and reactome gene sets were explored. Functional differences between all regions of interest were analyzed and expansive tumor parts (A), central tumor parts (B) and central stromal areas (C) were used as respective references. ● Hallmark gene sets, ● Reactome gene sets.
